# Vitamin D and Breast Cancer: Should Our Patients Be Taking a Supplement?

**DOI:** 10.6004/jadpro.2017.8.1.1

**Published:** 2017-01-01

**Authors:** Pamela Hallquist Viale

Breast cancer remains a formidable disease in women. In 2017, the American Cancer Society notes that the estimated number of new female invasive breast cancer cases is 252,710, with an estimated 40,610 women dying of the disease (American Cancer Society, 2017). Previously published studies have reported on possible associations between vitamin D levels and breast cancer, yet the data have sometimes appeared conflicted. Many of these studies examined breast cancer incidence and vitamin D, but few of them looked at survival rates and vitamin D levels. High levels of vitamin D have been reported to affect survival in women with premenopausal women; however, the actual role of this vitamin and the risk of breast cancer continues to be debated.

## PREVIOUS RESEARCH

A meta-analysis published in 2014 revealed that high serum 25(OH)D was associated with a lower mortality rate in patients with breast cancer (Mohr, Gorham, Kim, Hofflich, & Garland, 2014). The researchers examined five studies assessing the relationship between 25(OH)D and mortality in breast cancer, with results demonstrating that higher serum concentrations were associated with lower case-fatality rates after diagnosis of the disease. Patients in the highest quantile of 25(OH)D demonstrated half the death rate from breast cancer compared with those in the lowest quantile. The researchers in this case concluded that all patients with breast cancer should be monitored for serum 25(OH)D levels and that those levels should be returned to a normal range (30–80 ng/mL).

An earlier study in 2009 by Goodwin and colleagues examined 512 women with early breast cancer and their vitamin D levels (Goodwin, Ennis, Pritchard, Koo, & Hood, 2009). Women with deficient vitamin D levels had an increased risk of distant recurrence compared with those participants demonstrating sufficient levels; the researchers concluded that vitamin D deficiency may be associated with poorer outcomes in breast cancer. More recently, a study published in *Endocrinology* reported that ablation of vitamin D receptor expression within breast cancer cells may speed up primary tumor growth, contributing to metastasis (Williams et al., 2016).

## CURRENT DATA

The most recent study to look at vitamin D levels and mortality is by Yao and colleagues, published in the November 2015 issue of *JAMA Oncology*. Researchers reported on a prospective cohort study of 1,666 women with breast cancer with ongoing follow-up. Participants with a diagnosis of incident invasive breast cancer were enrolled in the study within 2 months of their diagnosis. The mean age of the patients was 58.7 years.

The results demonstrated that serum 25(OH)D levels were lower in women with advanced-stage tumors and lowest in premenopausal women with triple-negative tumors (Yao, Kwan, & Ergas, 2016). Women with the highest levels had a superior overall survival (OS) compared with those with lower levels, even after adjustment for clinical prognostic factors. The association with OS was stronger with premenopausal women; associations were found with breast cancer–specific survival and invasive disease–free survival as well (OS: hazard ratio [HR], 0.45; 95% confidence interval [CI], 0.21–0.96; breast cancer–specific survival: HR, 0.37; 95% CI, 0.15–0.93; invasive disease–free survival: HR, 0.58; 95% CI, 0.34–1.01; all after full adjustment). Premenopausal women with breast cancer and the highest vitamin D levels were 55% more likely to survive their disease. These findings are compelling given the prospective nature of the study design and the large number of participants.

## ROLE OF THE ADVANCED PRACTITIONER

The data above reflect examples of current research on breast cancer mortality and vitamin D levels. The final answer regarding the role of vitamin D levels and breast cancer is not known, but as advanced practitioners caring for this population of patients, we must take note of current study results and make appropriate recommendations to our patients. Vitamin D deficiency appears to play a role in the outcomes of women with invasive breast cancer, particularly those with premenopausal disease. Vitamin D deficiency remains a problem in the general population (Pilz et al., 2016). It is appropriate to recommend a healthy lifestyle with balanced sunlight exposure; however, supplementation is usually required to increase levels of vitamin D in the general population (Pilz et al., 2016).

For patients with established breast cancer diagnoses, the answers are not completely known; however, experts suggest adhering to the recommendations of the Institute of Medicine (600 units of vitamin D a day for people under age 70 and 800 units for people over age 70). Further research is needed to more firmly establish the role of vitamin D in patients with breast cancer, but current study results are persuasive. If higher serum levels of 25(OH)D can contribute to improved outcomes in patients with breast cancer, supplementation would seem to be an easy treatment to implement.
